# Tick-borne tularaemia as a differential diagnosis in ulcerative skin infection and regional lymphadenopathy in children: a case report

**DOI:** 10.1186/s13256-026-06491-0

**Published:** 2026-08-14

**Authors:** Piet Erik Beusker, Karen Schonhofen, Kerstin Landwehr, Eckard Hamelmann, Janina Soler Wenglein

**Affiliations:** Department of Pediatrics, University Hospital OWL, Protestant Hospital of the Bethel Foundation, Bielefeld University, Bielefeld, Germany

**Keywords:** Tularaemia, Francisella tularensis, Arthropods, Tick bite, Tick-borne, Ulceroglandular

## Abstract

**Background:**

Tularaemia is a rare but re-emergent zoonosis in Germany, which reached a new peak in incidence in 2024. The infection rate of ticks in Germany with *Francisella tularensis* is only about 8% and the transmission of a clinically apparent infection by blood-sucking arthropods such as ticks, mosquitoes or horseflies accounts for about 30% of the infection routes in humans. Arthropod-borne tularaemia mainly presents as the glandular or ulceroglandular form. Delayed diagnosis and initiation of treatment with associated increased risk of complications are common. Those complications may include necrotizing abscess formation in up to 65% of cases. To our knowledge, only two cases of tularaemia following tick bites in children in Germany have been published to date. Both report cases of glandular tularaemia. The present case is therefore the first report of a clinically compatible, serology-supported presumptive case of ulceroglandular tularaemia following a tick bite in a child in Germany. It underscores the increasing relevance of tularaemia as a differential diagnosis in ulcerative skin lesions with regional lymphadenopathy.

**Case presentation:**

In the present clinical case, a 3-year-old white German boy presented with ulcerative skin infection, regional lymphadenopathy and fever following a tick bite. Calculated treatment with intravenous ampicill in and clindamycin as well as topical antiseptics initially did not result in lasting clinical improvement. In the course of typical clinical symptoms and indirect pathogen detection by serology, ulceroglandular tularaemia was assumed and appropriate oral treatment with ciprofloxacin was initiated, under which the symptoms resolved fast and completely.

**Conclusion:**

The combination of increasing incidence rates, often delayed diagnosis, and therefore high complication rates underscores the clinical relevance of this case, which highlights the growing importance of tularaemia as a differential diagnosis in ulcerative skin lesions with regional lymphadenopathy.

## Background

### Epidemiology

Tularaemia, caused by *Francisella tularensis* and in Europe mainly by the subspecies *holarctica*, is a rare but re-emergent zoonosis in Germany, Europe and the United States [1–4]. Since 2001, the Robert Koch Institute (RKI) has recorded an almost continuous increase in the number of infections from < 10 to a peak of 214 cases/year in Germany in 2024 [5, 6]. This trend is congruent with the European surveillance data from the European Center for Disease Prevention and Control (ECDC) and from the Centers for Disease Control and Prevention (CDC), United States [3, 4]. However, the actual incidence is likely to be significantly higher due to often non-specific symptoms and limited diagnostics. In Germany, the majority of transmission cases occur in the southern federal states [5].

### Microbiology

*Francisella tularensis* itself is a gram-negative, obligate aerobic and facultative intracellular bacterium [1]. The most common routes of transmission of *F. tularensis* to humans are oral ingestion of contaminated animal food, direct contact with infected animals or animal material, and transmission through the bites of blood-sucking arthropods such as ticks, mosquitoes or horseflies [7, 8]. Another, less common transmission route is the inhalation of aerosols containing pathogens [7, 8]. In a long-term survey from the highest incidence area in Germany, Baden-Württemberg, the transmission rate is stated to be up to 30%, of which around 65% is caused by tick bites [9]. In Central Europe, *Ixodes ricinus* and *Dermacentor reticulatus*, among others, have been described as vectors [1]. The infection rate with *F. tularensis* in domestic ticks itself was reported 8.4% in a German study [10].

### Clinical presentations

Incubation period of tularaemia usually is 3–5 days but can range from 1–14 days. The initial symptoms are non-specific and often include fever, malaise, chills and headache. Less commonly are cough, myalgia, shortness of breath, abdominal pain, sore throat or following skin rashes. In the case of transmission by penetration into the skin, the first lesion appears as a cutaneous ulcer, which is usually solitary and develops into an encrusted, ulcerated or pustular wound as the disease progresses [8].

Several forms of tularaemia are distinguished based on the site of entry of the bacterium The glandular form is the most common clinical presentation (29.7%) and is characterized by lymphadenitis without a visible primary lesion. Ulceroglandular tularaemia is the second most common form of clinical presentation (15.6%) and appears after transmission of the infection via the skin or mucous membranes. At the site of pathogen-entry, a primary maculopapular lesion later develops into an ulcer associated with localized lymphadenitis. Less common, pulmonary tularaemia (12.2%) can occur after inhalation of aerosols containing the pathogen. In its maximum variant, this can manifest as bronchopneumonia with coughing, chest pain, breathing difficulties, sweating, nausea, vomiting and swelling of the hilar lymph nodes. The even rarer oropharyngeal form (5.3%) develops after the ingestion of contaminated food or water and leads to symptoms such as tonsillitis, pharyngitis or stomatitis with the risk of developing a retropharyngeal abscess. Intestinal tularaemia (4.6%) manifests itself with diarrhea, vomiting or abdominal pain. Another rare form of tularaemia is caused by inoculation of bacteria into the conjunctiva, which leads to painful oculoglandular tularaemia (1.8%) with unilateral conjunctivitis, localized lymphadenopathy, pronounced swelling of the eyelids, photophobia and purulent secretion. Finally, there is a typhoidal form (1.1%), which presents with general symptoms of the disease without any localized findings [1, 8].

Tularaemia is associated with a relevant rate of complications. Lymph node abscesses develop in up to 60–65% of patients with tularaemia-associated lymphadenopathy [11–13]. However, this cannot necessarily be considered a treatment failure, as many of these patients are diagnosed and treated late, when the abscess has already occurred. This is reflected by observations from everyday clinical practice where the median latency from first clinical symptoms to diagnosis was 30 (19–54) days [1]. In a cohort study of 145 patients, Meric et al. were able to show that antimicrobial therapy which was only started 14 days after the onset of the first symptoms significantly reduced the success rate and prolonged the recovery time [14]. These results indicate the pivotal relevance of early diagnosis and fast initiation of treatment to avoid the frequent treatment failures with complications.

Tularaemia in children in Germany is rare: To our knowledge, only two cases have been published to date. Both papers report on pediatric patients (aged 14 and 2 years, respectively) who developed regional lymphadenopathy without persistent skin lesions after a tick bite, which was later diagnosed as glandular tularaemia [15, 16]. To our knowledge we report the first presumed case of ulceroglandular tularaemia following a tick bite in a child in Germany. This underscores the relevance of this case, which points to the increasing relevance of tularaemia as a differential diagnosis in ulcerative skin lesions with regional lymphadenopathy.

## Case presentation

### Patient information

In May 2025, a 3-year-old white German boy without any medical or psychosocial pre-existing conditions accompanied by his mother presented to the Children's Emergency Room. The family history was reported to be negative for any chronical or severe medical conditions. The mother had noticed a tick bite on the right lateral upper arm 5 days ago and emphasized that the child’s father is a hunter. The bite lesion now showed a circumscribed erythematous local reaction with a diameter of approximately 1 cm and pustular ulceration around the hypostome. In addition, the boy had developed fever and pronounced fatigue with reduced general condition and flu-like symptoms since the previous day. No other significant diseases were mentioned in the previous history; a scarlet fever infection 2 weeks earlier with corresponding exanthema had been treated with antibiotics for a total of 7 days up to the day of presentation.

### Clinical findings

**Fig. 1:**
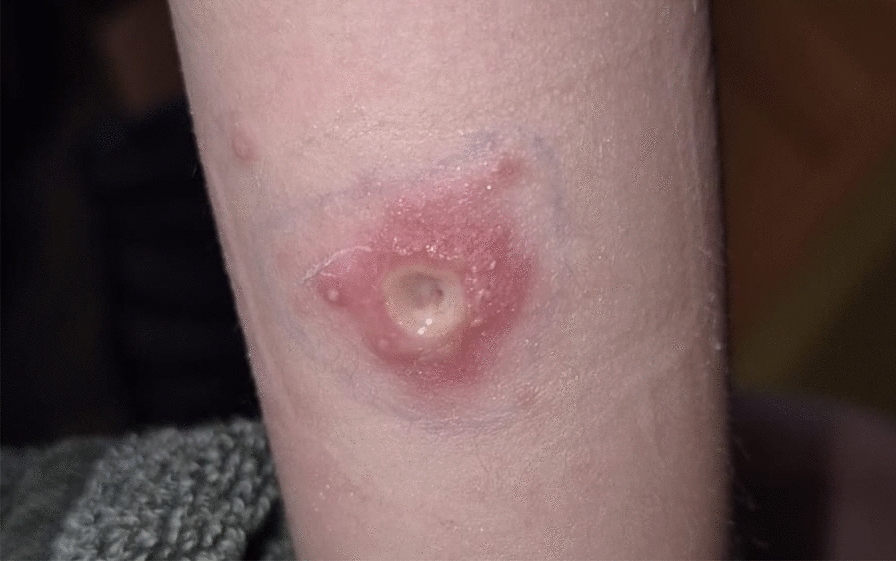
Local findings on the right upper arm 15 days after tick bite. The image shows the typical skin findings with raised, secreting ulceration. Accompanying regional lymphadenopathy is common and defines the ulcerative-glandular manifestation of tularaemia

The clinical examination revealed the local findings described above on the right upper arm (see Fig. 1), as well as a displaced, pressure-dolent lymph node on the right axilla with a diameter of approximately 1.5 cm. Further clinical examination findings were unremarkable.

### Timeline

A timeline is provided in Table 1.

**Table 1:** Timeline

Week	Clinical course
1	Tick-bite, clinical symptoms including fever, admission to hospital, start of Ampicillin i. v
2	Addition of Clindamycin i. v., fever reduction, switch to Amoxicillin p. o., discharge
3	End of Amoxicillin p. o
8	Follow-up with remaining local symptoms, extended serological tests
9	Suspected diagnosis of tularaemia, start of Ciprofloxacin p. o
10	End of Ciprofloxacin p. o

### Diagnosic assessment

Laboratory chemistry revealed a C-reactive protein (CRP) of 40.4 mg/l (< 5 mg/l) and a leukocytosis of 18.3/nl, with no other abnormalities. The blood culture showed no evidence of germs during the hospital stay. The abdominal and thoracic sonography showed no pathological abnormalities apart from a right axillary lymph node swelling with a sonographically determined diameter of approx. 1.8 cm with no indication of malignancy or fusion. There was no serological evidence of an acute or previous Borrelia infection.

### Therapeutic intervention

On admission, we performed manual extraction of the hypostome and thorough topical antiseptic treatment with polihexanide and decylenamidopropyl betaine-containing solution. Assuming a bacterial infection after a tick bite with persistent, pronounced fever attacks and reduced general condition, we started a calculated intravenous antibiotic therapy with ampicillin (100 mg/kg/d) on the second day of hospitalization, initially suspecting a superinfected local tick bite and intending to also adress *Borrelia* using the prescribed antibiotic therapy. We escalated our antibiotic therapy to the combination of intravenous ampicillin and clindamycin (40 mg//kg/d) on the fourth day of treatment to improve coverage of *Staphylococcus aureus* as well as anaerobes and to reduce potential toxin production due to a lack of improvement of the patient’s clinical condition. After 5 days, the patient recovered from fever, experienced significant improvement in his general condition, with CRP decreasing to 7.2 mg/l and leukocytes returning to normal levels. The patient was discharged for outpatient continuation of antibiotic therapy with oral amoxicillin (750 mg/d) for an additional 5 days.

### Follow-up and outcomes

At the follow-up examination after discharge, the patient did not show complete regression of the ulcer. Therefore. a sample was sent for serological testing for tularaemia, which resulted in positive detection of *F. tularensis* IgG using Enzyme-linked immunosorbent assay (ELISA). At that point, collecting material for direct pathogen detection via PCR no longer seemed appropriate, since although the lesion was still clearly present and inflammatory, it was superficially closed. Although this provided only a single-time, indirect detection of the pathogen, we started further antibiotic treatment with oral ciprofloxacin (30 mg/kg/d) for another 10 days according to international treatment guidelines for *F. tularensis* [4]. This seemed to us to be the most sensible choice, as treatment with the alternatively recommended aminoglycosides – which are available only intravenously – did not appear appropriate given the moderate severity of the illness. Treatment with oral doxycycline would also have been possible, but would have required a significantly longer course of therapy [4]. The chosen treatment resulted in a gradual regression of the local findings and the general fatigue and finally in the complete recovery of the patient. No adverse events were reported.

In summary, we assumed a clinically compatible, serology-supported presumptive diagnosis of tick-borne tularaemia even though we did not collect a second serological test. We deliberately chose not to perform another diagnostic blood test because we did not want to further delay treatment, and a purely diagnostic blood test with no implications for treatment did not seem justified in the case of a 3-year-old child. In addition to common soft-tissue infections caused by streptococci or staphylococci, other differential diagnoses include rarer conditions such as rickettsiosis or anthrax; however, based on a comprehensive review of all findings, we considered these to be less likely.

## Discussion and conclusions

The present case is a clinically compatible, serology-supported presumptive case of ulceroglandular tularaemia following a tick bite in a child in Germany. A similar case was only documented in France in 2011, in which a 4-year-old boy developed ulceroglandular tularaemia after a tick bite on the head [17].

In routine clinical care in Germany, serology is the most common method for confirming the diagnosis of tularaemia (66.2%); direct pathogen detection using culture (15.6%) or PCR (22.1%) is much less common [1]. On the one hand, this is primarily due to the low sensitivity of cultivation, which is attributable to the short routine incubation period usually used in clinical laboratories, but insufficient for the detection of *F. tularensis*. In the present case, the initially cultured wound swab, which had been incubated according to standard methods, did not yield any evidence of the pathogen. On the other hand, pathogen detection using PCR is less common, as at the time of initial diagnosis, when the cutaneous ulcers are still open and produce secretions, tularaemia is often not considered as a differential diagnosis and therefore no pathogen-specific PCR test is requested. In the present case, the cutaneous ulcer was already closed and no longer secreting when tularaemia was first considered as a differential diagnosis. The diagnosis of tularaemia in medical care is therefore still mainly based on serological tests. The WHO defines tularaemia as presumptive if, in addition to the typical clinical symptoms, at least one positive serology is present and it is considered confirmed if there is at least a fourfold increase in titer or seroconversion in two serologies taken at different times [18]. In the present case, the diagnosis of tularaemia based on the typical picture of an ulcreroglandular form of presentation and an increased antibody titer of 20 days after tick bite must therefore be classified as presumptive but must not be formally regarded as confirmed. In numerous case series or cohort studies, however, the diagnosis of tularaemia has been defined analogously to the present case [11, 13, 14].

In children, diagnosis of tularaemia is also often delayed, as symptoms such as fever, local lymph node swelling and skin lesions can easily be confused with other, more common pediatric infections. A case series from France showed a median latency from first clinical symptoms to diagnosis of 23.5 days in 94 children [19]. For adults, this is even stated as up to 30 days [1]. However, particularly in view of the described multiple complications due to necrotizing abscess formation with frequent consecutive need for surgery due to delayed treatment, early diagnosis and start of appropriate antibiotic treatment with ciprofloxacin or doxycycline as soon as the diagnosis is suspected is of great importance. The combination of frequently delayed diagnosis in childhood and the rarity of the transmission route therefore underlines the clinical relevance of this case.

Finally, it remains unclear why the patient in this case initially showed partial remission with fever reduction under treatment with ampicillin and clindamycin. Possible explanations could be onset of control of the pathogen by the patient's own immune system despite insufficient antibiotic therapy, as well as the anti-inflammatory effects of clindamycin. The fact that both the cutaneous ulcer and the lymphadenopathy only completely resolved under treatment with ciprofloxacin underscores the importance of an accurate and fast diagnosis and initiation of the appropriate therapy.

## Limitations

First, in this case, no direct detection of the pathogen was achieved using culture or PCR, and only a single serological result is available; therefore, according to WHO criteria, the diagnosis must be classified as presumptive but not confirmed. Second, other relevant but less likely differential diagnoses, such as rickettsiosis and anthrax, were not ruled out by specific diagnostic tests.

The information from the patient’s history that our patient’s father is a hunter could have led to earlier initiation of the relevant diagnostic tests and, ultimately, possibly to a definitive diagnosis. However, given the clinical course and the good response to targeted therapy, we conclude that the single serological test result was sufficient to initiate treatment in this case.

## Data Availability

Not applicable.

## Abbreviations

CDC: Centers for Disease Control and Prevention
CRP: C-reactive protein
ECDC: European Center for Disease Prevention and Control
EFSA: European Food Safety Authority (EFSA) and European Centre for Disease Prevention and Control (ECDC), 2024)
ELISA: Enzyme-linked immunosorbent assay
*F. tularensis*: *Francisella tularensis*
PCR: Polymerase chain reaction
RKI: Robert-Koch-Institut
WHO: World Health Organization
